# Value of Annual MRI Monitoring in Patients With Multiple Sclerosis Treated With Ocrelizumab: A Number Needed to Scan Analysis

**DOI:** 10.1212/WNL.0000000000218379

**Published:** 2026-07-29

**Authors:** Lisa Schoof, Rick Heijnen, Laura Hogenboom, Zoé Léonie Elise Van Kempen, Brigit Alette de Jong, Bob W. Van Oosten, Joep Killestein, Frederik Barkhof, Eva M. Strijbis

**Affiliations:** 1 MS Center Amsterdam, Amsterdam UMC location Vrije Universiteit Amsterdam, Neurology, the Netherlands;; 2 MS Center Amsterdam, Amsterdam UMC location Vrije Universiteit Amsterdam, Radiology and Nuclear Medicine, the Netherlands; and; 3 UCL London, Institutes of Neurology and Healthcare Engineering, London, United Kingdom.

## Abstract

**Background and Objectives:**

Current guidelines recommend annual MRI scans during treatment with disease-modifying therapies, including ocrelizumab, in patients with multiple sclerosis (MS) to monitor treatment effectiveness and safety. However, the low rate of radiologic disease activity during long-term ocrelizumab treatment suggests that this recommendation should be re-evaluated to reduce burden on ocrelizumab-treated patients and the healthcare system. We aimed to describe the frequency of radiologic follow-up and the occurrence of radiologic disease activity in a cohort of ocrelizumab-treated MS patients to provide insight into the clinical value and efficiency of annual MRI monitoring in routine practice.

**Methods:**

We included ocrelizumab-treated MS patients from the Amsterdam MS Cohort, who had at least 2 brain MRI scans performed during ≥3 months of treatment. MRI scans were performed as part of routine clinical care and clinical MRI reports issued by radiologist were used. Radiologic activity was defined as either ≥1 contrast-enhancing lesions on a MRI scan performed ≥3 months after ocrelizumab initiation or any new/enlarging T2 lesions on a subsequent MRI scan compared with a scan performed at least ≥3 months after ocrelizumab initiation. The number needed to scan (NNS) was calculated as the inverse of the predicted probability of radiologic disease activity as a function of treatment duration in months, estimated using a mixed-effects logistic regression model.

**Results:**

A total of 342 patients (mean age at start ocrelizumab 40.3 years (SD 10.8); 65.8% female; 208 relapsing-onset MS and 41 primary progressive MS) were included, contributing 1,639 follow-up MRI scans. Radiologic disease activity was detected in 29 scans in 23 patients (1.8% of scans; 6.7% of patients) over a median treatment duration of 49 months. The NNS increased with treatment duration, especially after 24 months of treatment, reaching 81.3 at month 30 and 152.9 at month 36.

**Discussion:**

Routine annual MRI monitoring in patients with asymptomatic ocrelizumab-treated MS never led to treatments changes. The increasing NNS over time, especially after 2 years of treatment, indicates that MRI intervals can be safely extended in patients with stable MS treated with ocrelizumab, potentially doubling the interval from treatment year 2 onward (i.e.,; rebaseline, year 1, year 2, year 4, and year 8, etc.).

## Introduction

Anti-CD20 therapies are widely used as high-efficacy treatment (HET) in multiple sclerosis (MS). Currently, ocrelizumab, ofatumumab and ublituximab are registered for treatment of MS,^1^ and rituximab is often prescribed off-label.^2-4^ Recently, anti-CD20 therapies are more frequently initiated as first-line treatment.^5-8^ Among these agents, ocrelizumab holds a prominent position as the only anti-CD20 therapy approved for both relapsing-onset MS (RMS) and primary progressive MS (PPMS), with widespread use in clinical practice.

Radiologic monitoring of disease activity with MRI scans of the brain is an important component of the follow-up of patients receiving disease-modifying therapy (DMT), including ocrelizumab treatment, either for the detection of (asymptomatic) disease activity or side-effects of treatment. Current guidelines recommend annual brain MRI monitoring during treatment to assess treatment effectiveness and monitor safety.^9,10^ The MAGNIMS-CMSC-NAIMS guideline states that in clinically stable patients longer intervals can be considered after the first few years of treatment; however, it provides no specific recommendations on when such interval extension is appropriate or how long these intervals should be.^9^ In addition, apart from progressive multifocal leukoencephalopathy (PML) screening in JC-virus positive patients on natalizumab, no DMT-specific recommendations are given.^9^

Clinical trials have shown a low frequency of radiologic disease activity during ocrelizumab treatment, particularly with longer treatment duration.^11,12^ This raises questions about the value of annual MRI monitoring during long-term ocrelizumab treatment, especially given its burden on patients and costs to the healthcare system. On the other hand, missing radiologic disease activity may have important clinical consequences, as a treatment switch to, for example, autologous hematopoietic stem cell transplantation (aHSCT), can be indicated solely based on new radiologic disease activity, even in the absence of clinical symptoms, according to the most recent guidelines.^13^

Considering these arguments, we aimed to describe the frequency of radiologic follow-up and the occurrence of radiologic disease activity in a cohort of patients with MS treated with ocrelizumab to provide insight into the value and efficiency of annual MRI monitoring in routine clinical practice.

## Methods

### Participants

We included patients from the Amsterdam MS cohort, which is a prospective, observational registry that includes adult patients with all MS subtypes from the MS Center Amsterdam, a tertiary referral center in the Netherlands. Patients are followed longitudinally as part of routine clinical care; there are no additional inclusion or exclusion criteria for participation in the cohort itself.

For this analysis, we included patients from the Amsterdam MS cohort who met the following criteria: (1) an MS diagnosis according to the 2017 revisions of the McDonald criteria,^14^ (2) treatment with ocrelizumab, and (3) at least 2 brain MRI scans performed after a minimum of 90 days (3 months) of ocrelizumab treatment. Ocrelizumab treatment was defined as a recorded ocrelizumab start date without a registered stop date; therefore, patients with temporary treatment interruptions (e.g., due to pregnancy or intercurrent infections) were included, provided that there was no documented intention to permanently discontinue therapy. Treatment duration was calculated as the time between the first and last ocrelizumab infusion. Patients who had previously received another anti-CD20 therapy were excluded.

### Clinical and Radiologic Data

Data were extracted in September 2025 from the Amsterdam MS cohort registry, including clinical symptoms and other clinically relevant findings, such as possible pharmacovigilance-related events. All MRI scans were performed as part of routine clinical care. The indication for MRI scans is determined by the treating neurologist as part of routine clinical care, typically for annual disease monitoring or in response to new or worsening symptoms. In general, brain MRI scans are performed, while spinal cord imaging is reserved for specific clinical indications (e.g., new spinal symptoms). Standard clinical care MRI brain protocol consists of at least a FLAIR and PD-T2 sequence; gadolinium is not routinely administered. MRI reports issued by radiologist as part of routine clinical care were used for this analysis; scans were not re-reviewed. The presence of new or enlarged T2 lesions and contrast-enhancing lesions (CELs) was extracted from these routine reports.

### Definition Radiologic Disease Activity

Radiologic disease activity was defined as either ≥1 CEL on a MRI scan performed ≥3 months after ocrelizumab initiation or any new or enlarged T2 lesion on a subsequent MRI scan compared with a reference scan performed at least ≥3 months after ocrelizumab initiation.

### Statistical Methods

The number of MRI scans required to detect one scan with radiologic disease activity was calculated as the inverse of the predicted probability of radiologic disease activity (number needed to scan, NNS = 1/predicted probability). The predicted probability of radiologic disease activity as a function of treatment duration in months was estimated using a mixed-effects logistic regression model, which included radiologic disease activity on brain MRI as the binary outcome, time since ocrelizumab initiation (in months) as a continuous fixed effect, and a random intercept for each patient to account for repeated measures. Analyses were based on available data only. Statistics were conducted using R version 4.3.2.

### Standard Protocol Approvals, Registrations, and Patient Consents

This study includes data from the Amsterdam MS cohort, a registry approved by the ethics board of the Amsterdam UMC (2020-269). All patients provided written informed consent for participation as per protocol of the Amsterdam MS cohort.

### Data Availability

Data are available from the corresponding author on reasonable request.

## Results

### Patient Characteristics

A total of 342 patients with ocrelizumab-treated MS, 301 patients with RMS and 41 patients with PPMS, were included. The median disease duration at ocrelizumab initiation was 86 months (interquartile range [IQR] 29–160.5) for patients with RMS and 19 months (IQR 4.5–66.5) for patients with PPMS. Patients received a median of 8 full ocrelizumab cycles (IQR 5–11.5) for RMS and 10 cycles (IQR 7–12.5) for PPMS over a median treatment duration of 48 months (IQR 30–71) and 56 months (IQR 36.5–73), respectively. The median treatment interval between 2 full ocrelizumab cycles was 6.2 months (189 days, IQR 182–197). In total, 71 patients had an interval between 2 infusions longer than 12 months, of whom 21 had an interval longer than 18 months. The longest treatment interval was 26 months (793 days). On the last MRI scan before ocrelizumab initiation 242 (70.8%) of the patients showed radiologic disease activity (69% for the patients with RMS and 85.4% for the patients with PPMS). Detailed patient characteristics are summarized in Table 1.

**Table 1:** Patient Characteristics

Characteristics	All patients (N = 342)	RMS patients (n = 301)	PPMS patients (n = 41)
Women, n (%)	225 (65.8)	208 (69.1)	17 (41.5)
Age at start OCR, y, mean ± SD	40.3 ± 10.8	38.9 ± 10.3	51 ± 7.9
BMI, kg/m^2^, mean ± SD	21.5 ± 4.2	21.4 ± 4.1	21.9 ± 4.5
Disease duration at start OCR, mo, median (IQR)	79 (20.8–143)	86 (29–160.5)	19 (4.5–66.5)
Treatment duration, mo, median (IQR)	49 (31–72)	48 (30–71)	56 (36.5–73)
Number of OCR cycles, median (IQR)	8 (6–12)	8 (5–11.5)	10 (7–12.5)
Total number of MRI (brain or brain/spine) scans during OCR treatment, median (IQR)	4 (3–6)	4 (3–6)	4 (3–5.5)
Treatment interval between 2 full OCR cycles in days, median (IQR)	189 (182–197)	189 (182–197)	189 (182–196)
Disease activity on last MRI before OCR initiation, n (%)	242 (70.8)	207 (69)	35 (85.4)
CELs on last MRI before OCR initiation, n (%)	104 (30.4)	93 (31)	11 (26.8)
Last DMT before OCR initiation, n (%)			
None	83 (24.3)	47 (15.6)	36 (87.8)
Interferon	10 (2.9)	10 (3.3)	—
Glatiramer acetate	27 (7.9)	25 (8.3)	2 (4.9)^a^
Teriflunomide	16 (4.7)	16 (5.3)	—
Dimethyl fumarate	95 (27.8)	94 (31.2)	1 (2.4)^a^
Daclizumab	5 (1.5)	5 (1.7)	—
Mitoxantrone	2 (0.6)	2 (0.7)	—
Fingolimod	40 (11.7)	38 (12.6)	2 (4.9)^a^
Ozanimod	5 (1.5)	5 (1.7)	—
Cladribine	1 (0.3)	1 (0.3)	—
Alemtuzumab	1 (0.3)	1 (0.3)	—
Natalizumab	57 (16.7)	57 (18.9)	—

Abbreviations: BMI = body mass index; CELs = contrast-enhancing lesions; DMT = disease-modifying therapy; IQR = interquartile range; OCR = ocrelizumab; PPMS = primary progressive MS; RMS = relapsing MS.

a Glatiramer acetate and dimethyl fumarate were prescribed in PPMS patients when disease course was unclear. Fingolimod was prescribed in PPMS patients in a clinical trial.

### MRI Characteristics

A total of 1,639 MRI scans were performed during ocrelizumab treatment; 1,433 brain MRI scans, 187 combined brain/spinal cord MRI scans, and 19 spinal cord-only MRI scans. Among the first MRI scans, 31.6% (108/342) were performed between 3 and 6 months after ocrelizumab initiation, with a median time to the first MRI scan of 5.1 months (156.5 days; IQR 84.8–276.5). Of all first follow-up MRI scans, 142 (41.5%) were performed with contrast administration.

### Radiologic Disease Activity During Ocrelizumab Treatment

**Figure 1:**
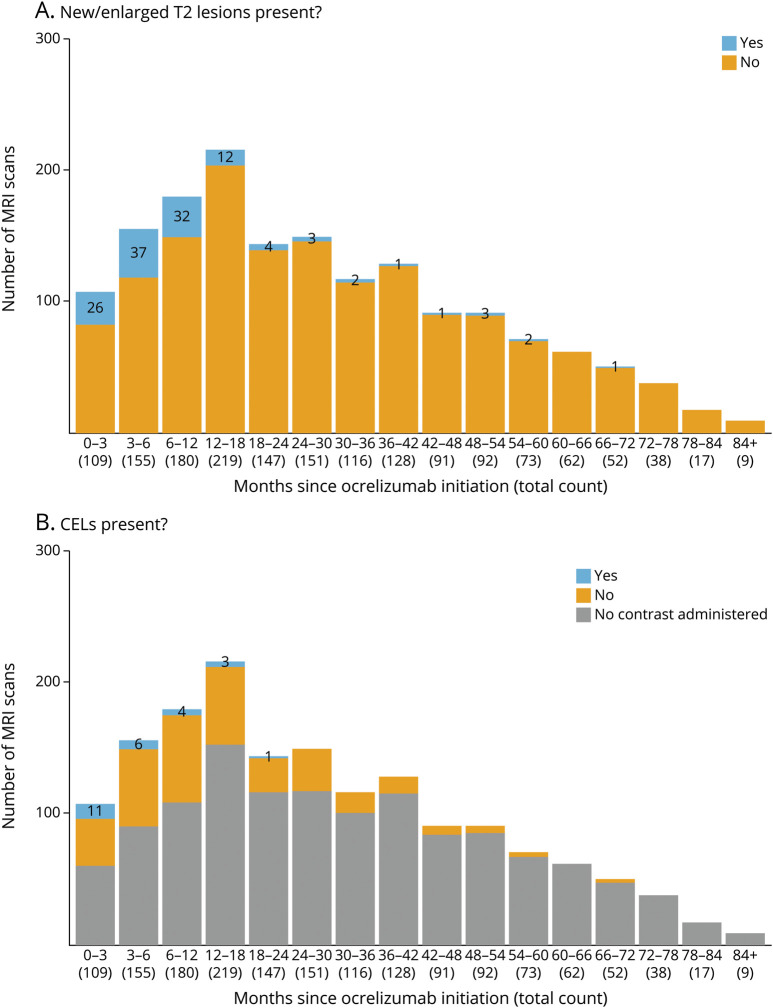
MRI Scans Showing New/Enlarged T2 Lesions or CELs During Ocrelizumab Treatment CELs = contrast-enhancing lesions.

The number of MRI scans showing new T2 lesions or CELs by months since ocrelizumab initiation is shown in Figure 1.

**Figure 2:**
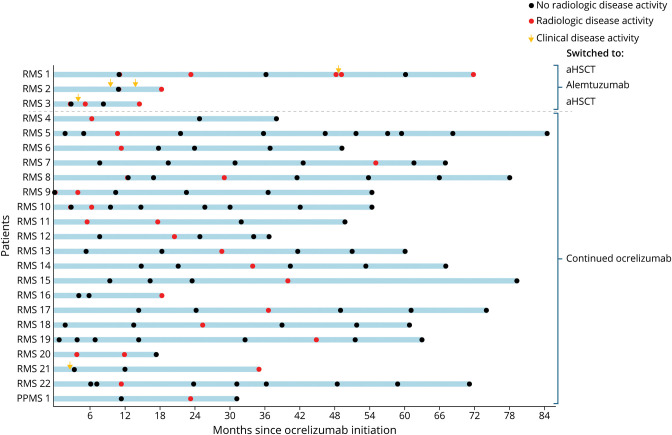
Individual Patients With Radiologic Disease Activity During Ocrelizumab Treatment aHSCT = autologous Hematopoietic Stem Cell Transplantation; PPMS = primary progressive MS; RMS = relapsing MS.

In total, radiologic disease activity was detected in 29 scans (1.8%) in 23 patients (6.7%). In 3 of these patients, disease activity prompted a switch in DMT; all of these patients also had concurrent clinical symptoms. Two patients (RMS1 and RMS3 in Figure 2) switched to aHSCT. One patient (RMS2) switched to alemtuzumab. For the remaining 20 patients, radiologic disease activity did not lead to a DMT switch and was generally minimal, nonrecurring and not accompanied by clinical symptoms. Among the patients who continued ocrelizumab, there was one patient (RMS21) who experienced clinical symptoms. On MRI, there were concurrent new T2 lesions, although these did not meet the criteria for radiologic activity. See also Figure 2.

Most radiologic disease activity was observed on brain MRI scans or on combined brain/spinal cord MRI scans. Although 4 spinal cord–only MRI scans showed new or enlarged T2 lesions, none met the criteria for radiologic disease activity. Among the combined brain/spinal cord MRI scans, 11 showed new or enlarged T2 lesions confined to the spinal cord; of these, 2 met the criteria for radiologic disease activity.

### Number Needed to Scan

**Figure 3:**
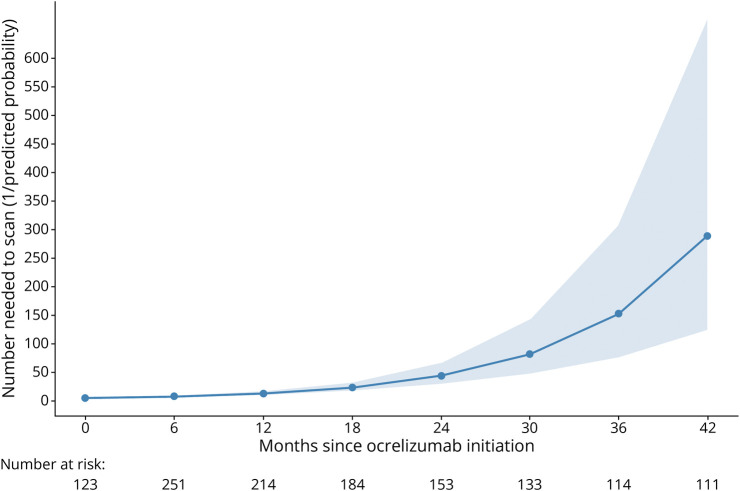
Number of MRI Scans Required to Detect 1 Scan With Radiologic Disease Activity (Number Needed to Scan) per Treatment Duration in Months Data are truncated at 42 months because beyond this time, the 95% confidence intervals widen substantially due to the low probability of disease activity, and fewer than 100 MRI scans contribute to each time point, reducing the reliability of the estimates.

The number of MRI scans required to detect one scan with radiologic disease activity, NNS, increased with treatment duration, indicating that the probability of detecting radiologic disease activity declines with longer treatment duration. At 3 months, the NNS was 5.6 (95% CI 4.3–7.2). This increased to 7.3 (95% CI 5.8–9.3) at 6 months, 12.9 (95% CI 10.1–16.4) at 12 months, 23.4 (95% CI 17.2–32.1) at 18 months, and 43.4 (95% CI 28.4–66.7) at 24 months. Beyond 24 months, the NNS increased substantially: 81.3 at 30 months (95% CI 46.6–142.3), 152.9 at 36 months (95% CI 76.2–307.8) and 288.3 at month 42 (95% CI 124.4–669.8). See also Figure 3 and eTables 1 and 2.

### Pharmacovigilance

In this cohort, except for 2 previously described cases of carry-over PML following the switch from natalizumab to ocrelizumab due to JC-virus positivity,^15^ no opportunistic infections or other pharmacovigilance-related abnormalities were observed on any of the MRI scans.

## Discussion

In this cohort of patients with MS treated with ocrelizumab in routine clinical practice, radiologic disease activity was found in 29/1,639 (1.8%) MRI scans in a total of 23/342 (6.7%) patients over a median treatment duration of 49 months (8 full cycles). Importantly, all patients who switched to another DMT due to radiologic disease activity showed concurrent clinical symptoms before the MRI scan where the disease activity was identified. Furthermore, no opportunistic infections or pharmacovigilance-related abnormalities were observed, indicating that routine MRI monitoring for safety purposes is unnecessary.

The observed low frequency of radiologic disease activity, that is consistent with data of clinical trials,^11^ suggests that annual MRI monitoring may not be necessary. This is further supported by the increasing NNS with treatment duration, especially with >2 years of ocrelizumab treatment (NNS 43.4 at 24 months, 81.3 at 30 months, 152.9 at 36 months, and 288.3 at month 42). In interpretating this NNS, it is important to note that patients who discontinued treatment due to disease activity (n = 3) were included in the NNS calculation but no longer after treatment discontinuation and contributed less follow-up time to the analysis. As a result, at later time points, the remaining cohort represents a lower-risk population and potentially biasing estimates toward lower detection rates and overestimating the NNS. In addition, with longer treatment duration less MRI scans contributed to each time point, widening the 95% confidence intervals and reducing the reliability of the estimates. After 42 months, less than 100 MRI scans contributed to the calculation.

Notably, only 16% of RMS patients were treatment-naive at ocrelizumab initiation, probably reflecting early prescribing patterns when ocrelizumab was mainly used as a second-line or third-line therapy. In recent years, however, its use has shifted to earlier disease stages, increasingly including first-line initiation, even in patients with lower baseline inflammatory disease activity. These patients often show lower rates of radiologic progression on treatment. Consequently, the NNS estimates in our study likely may not be fully generalizable to current clinical practice, where ocrelizumab is increasingly initiated earlier and in patients with lower baseline disease activity. In these first-line populations, the true NNS is likely to be even higher.

For the MRI results, the database relies on radiology reports from routine clinical practice where enlarged lesions are not assessed systematically and no formal definition is applied. As a result, enlarged lesions are only reported if the radiologist specifically looks for them or if they are noticed incidentally. Consequently, enlarged lesions may have been missed and this type of radiologic disease activity may be underreported in this cohort. Furthermore, routine radiology reports are based on visual assessments without the use of automated analyses, which may also contribute to an underestimation of radiologic disease activity and an overestimation of the NNS. In addition, MRI follow-up intervals and the administration of contrast were determined as part of routine clinical care and were not systematic. This may have reduced the sensitivity for detecting radiologic disease activity and contributed to an overestimation of the NNS.

Although in this cohort, routine annual MRI monitoring in asymptomatic patients never led to treatment changes, in clinical practice MRI activity in clinically stable patients may prompt a repeat MRI before treatment modification. Therefore, when interpreting our results, it should be considered that MRI activity detected in clinically stable patients does not necessarily lead to immediate treatment changes, as repeat imaging or additional diagnostics can be performed first.

Guidelines recommend performing a “rebaseline” MRI scan with contrast 3–6 months after treatment initiation; however, only 31.6% (108/342) of patients had a scan performed in this timeframe, and only 36.1% (39/108) of these included contrast. This highlights a gap between guideline recommendations and routine clinical practice, potentially due to logistical challenges, patient burden, or evolving practices.

Our cohort only included ocrelizumab-treated patients. Further research is needed to determine whether these findings can be generalized to other anti-CD20 therapies with similar mechanisms of action and low rates of radiologic disease activity.^16-18^

This cohort reflects routine clinical practice, including patients whose treatment interval were extended due to B cell–tailored ocrelizumab dosing, holidays, pregnancies, or side effects such as infections. This heterogeneity increases the generalizability of our findings, providing a realistic view of radiologic disease activity and MRI follow-up in routine care, beyond the controlled environment of clinical trials.

Routine annual MRI monitoring in asymptomatic patients never led to treatments changes. The increasing NNS over time, especially after 2 years of treatment, indicates that MRI intervals can be safely extended in stable patients treated with ocrelizumab; potentially doubling the interval from treatment year 2 onward (i.e.,; rebaseline, year 1, year 2, year 4, and year 8, etc.). Radiologic monitoring remains essential during early ocrelizumab treatment and in clinically symptomatic patients to identify cases requiring treatment re-evaluation.

## Glossary

CEL: contrast-enhancing-lesion
DMT: disease-modifying therapy
HET: high-efficacy treatment
IQR: interquartile range
MS: multiple sclerosis
NNS: number needed to scan
PML: progressive multifocal leukoencephalopathy
PPMS: primary progressive MS
RMS: relapsing-onset MS
